# Electrochemical Nitrogen Fixation for Green Ammonia: Recent Progress and Challenges

**DOI:** 10.1002/advs.202300951

**Published:** 2023-06-08

**Authors:** Haneul Jin, Suyeon S. Kim, Sandhya Venkateshalu, Jeseok Lee, Kwangyeol Lee, Kyoungsuk Jin

**Affiliations:** ^1^ Department of Energy and Materials Engineering Dongguk University‐Seoul Seoul 04620 Republic of Korea; ^2^ Department of Chemistry and Research Institute of Natural Science Korea University Seoul 02841 Republic of Korea

**Keywords:** ammonia production, catalyst design, electrochemical nitrogen reduction, lithium‐mediated, reaction mechanism

## Abstract

Ammonia, a key feedstock used in various industries, has been considered a sustainable fuel and energy storage option. However, NH_3_ production via the conventional Haber–Bosch process is costly, energy‐intensive, and significantly contributing to a massive carbon footprint. An electrochemical synthetic pathway for nitrogen fixation has recently gained considerable attention as NH_3_ can be produced through a green process without generating harmful pollutants. This review discusses the recent progress and challenges associated with the two relevant electrochemical pathways: direct and indirect nitrogen reduction reactions. The detailed mechanisms of these reactions and highlight the recent efforts to improve the catalytic performances are discussed. Finally, various promising research strategies and remaining tasks are presented to highlight future opportunities in the electrochemical nitrogen reduction reaction.

## Graphical abstract

This review discusses recent progress and challenges related to the electrochemical nitrogen reduction reaction. In addition, this review highlights promising research strategies and remaining tasks for future opportunities in electrochemical nitrogen reduction catalysis.

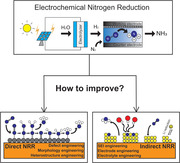

## 1 Introduction

**Figure 1:**
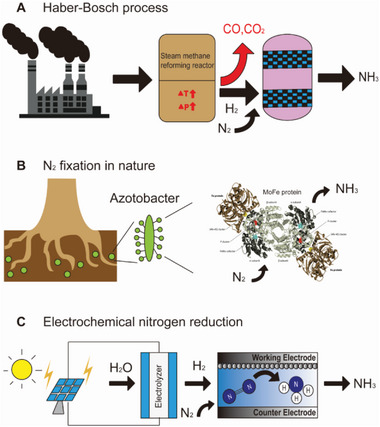
Nitrogen fixation. Three representative routes to produce ammonia from N_2_: A) Thermochemical Haber–Bosch. B) biological N_2_ fixation by nitrogenase enzyme and C) electrochemical nitrogen reduction system.

Artificial nitrogen fixation through the Haber–Bosch process, which was developed a century ago, is regarded as one of the most important technological pillars that support modern industrial society.^[^ ^1^, ^2^, ^3^, ^4^, ^5^, ^6^, ^7^ ^]^ Thus far, ammonia (NH_3_) has served as the key feedstock in various industries manufacturing fertilizers, pharmaceuticals, and textiles.^[^ ^5^, ^8^, ^9^, ^10^ ^]^ NH_3_ has recently emerged as a sustainable energy vector owing to facile liquefaction (−33.3 °C at atmospheric pressure or 7.5 bar at room temperature) and well‐established NH_3_ storage and transportation infrastructures.^[^ ^11^, ^12^, ^13^, ^14^ ^]^ However, the Haber–Bosch process is an energy‐intensive and expensive process requiring conditions of 300–550 °C and 250 bar with iron oxide catalysts, resulting in a substantial carbon footprint from CO_2_ production (**Figure** **1**A).^[^ ^15^, ^16^, ^17^, ^18^, ^19^ ^]^ In nature, an enzyme called nitrogenase can facilitate nitrogen reduction under ambient conditions.^[^ ^10^, ^16^, ^20^ ^]^ Although biological nitrogenases in diazotrophic microorganisms have inspired the design of synthetic catalysts for nitrogen fixation,^[^ ^21^, ^22^, ^23^, ^24^ ^]^ no catalytic system developed thus far has demonstrated a high level of selectivity or efficiency of nitrogenases (Figure 1B).

The electrochemical synthetic route for nitrogen fixation has received considerable attention as it can facilitate the green production of NH_3_ without the formation of harmful pollutants while using electricity generated from sustainable sources (Figure 1C).^[^ ^8^, ^25^, ^26^, ^27^, ^28^, ^29^, ^30^ ^]^ The electrochemical nitrogen reduction reaction (eNRR) proceeds as follows: 1) N_2_ molecules approach the electrode surface; 2) electrical charges are exchanged and protons are injected to break bonds and hydrogenate N_2_ (bond dissociation energy = 941 kJ mol^−1^); 3) NH_3_ is released.^[^ ^26^, ^31^ ^]^ The net nitrogen reduction reactions in acidic and alkaline electrolytes follow different reaction pathways but have the same equilibrium potential of 0.56 *V*_SHE_.^[^ ^32^, ^33^ ^]^

(1) $$N_{2}+6H^{+}+6e^{-}\to 2{\mathrm{NH}}_{3}\left(\mathrm{acidic}\;\mathrm{electrolyte}\;\mathrm{condition}\right)$$

(2) $$\begin{array}{ccc} N_{2}+6H_{2}O+6e^{-} & \to & 2{\mathrm{NH}}_{3}+6{\mathrm{OH}}^{-}\\ & & \left(\mathrm{alkaline}\;\mathrm{electrolyte}\;\mathrm{condition}\right) \end{array}$$

**Figure 2:**
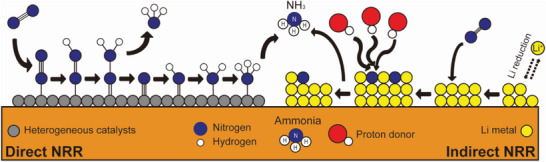
Schematic illustration of DNRR and INRR pathways. DNRR route shows the stepwise hydrogenation of the adsorbed N_2_ molecules on the electrode‐immobilized catalyst surface to produce ammonia. On the other hand, in INRR route, electroplating of lithium occurs on the electrode, followed by subsequent nitrogen splitting and hydrogenation on the Li surface.

There are several review articles that have summarized studies of electrochemical nitrogen reduction (eNRR).^[^ ^17^, ^34^, ^35^, ^36^, ^37^, ^38^, ^39^, ^40^, ^41^, ^42^, ^43^, ^44^, ^45^, ^46^, ^47^, ^48^, ^49^, ^50^ ^]^ In this review, we aim to classify recent eNRR studies into two distinct classes, and present the current mechanistic understanding of each pathway. Based on the reaction pathways (as shown in **Figure** **2**), studies on eNRR can be divided into two categories: direct nitrogen reduction reaction (DNRR) and indirect nitrogen reduction reaction (INRR). The use of electrons is the most significant difference between DNRR and INRR. The term Indirect was chosen for Li‐mediated pathway because electron is used to reduce “Li^+^” to metal Li, whereas electron in DNRR pathway is used to break and activate the “nitrogen” triple bond. DNRR refers to direct electrochemical nitrogen activation on a heterogeneous catalyst surface. In this pathway, heterogeneous catalysts only provide the binding sites for dinitrogen molecules. In contrast, INRR involves reactive metal species, such as Li, with a strong reducing ability to break triple nitrogen bonds (N≡N). While the electrons from the electrode are utilized to “directly” activate dinitrogen in the DNRR pathway, electrons in INRR are solely consumed to form Li metal films, which “indirectly” convert dinitrogen into lithium nitride. In other words, instead of the sequential activation of nitrogen on the catalyst surface, INRR harnesses metal nitride intermediates to produce NH_3_ from dinitrogen in a nonaqueous system. Although both selectivity (Faradaic efficiency (FE)) and reactivity (current density) in the INRR have been remarkably improved, the high energy input required to initiate Li plating on the electrode remains a considerable roadblock.

This review highlights the most recent progress in the two eNRR methods, DNRR and INRR. We aim to provide useful guidelines for future research by identifying the most significant developments and critical challenges. Initially, we focus on the reaction mechanisms of DNRR and INRR, based on the findings from electrokinetic, in situ, and ex situ spectroscopic, and various computational studies for eNRR. The effects of the catalyst design, including the morphology and heterostructure, on the DNRR are presented, considering the binding affinity of dinitrogen molecules on the catalyst surface during catalysis. The engineering strategies for the electrochemical components of INRR are discussed, followed by an overview of recent advances in Li‐mediated nitrogen reduction. Finally, we point to the most salient challenges currently associated with DNRR and INRR, suggesting potential research avenues to prompt research advances in this fledgling field.

## 2 Electrochemical Mechanisms for DNRR and INRR

The sluggish nitrogen reduction kinetics are primarily attributed to the thermodynamically stable triple N─N bond in N_2_, for which the bond (941 kJ mol^−1^) and ionization (15.6 eV) energies are considerably high.^[^ ^51^, ^52^, ^53^, ^54^ ^]^ Moreover, N_2_ has a negative electron affinity (−1.9 eV) and requires additional electrical energy for electron injection. For instance, the first protonation of *N_2_ to *NNH in the DNRR is endothermic (∆H^0^ = +37.6 kJ mol^−1^).^[^ ^11^, ^55^, ^56^ ^]^ Furthermore, electron excitation in N_2_ is significantly hindered by the large gap between the highest occupied and lowest unoccupied molecular orbitals: 10.82 eV (or 1044.0 kJ mol^−1^). Finally, compared with other gases, N_2_ has low solubility in aqueous solutions (0.6 mmol L^−1^ in water at room temperature). Herein, we present the recent understanding of the mechanism of each pathway.

### 2.1 DNRR (Catalyst‐Mediated eNRR)

#### 2.1.1 Proposed Mechanisms for DNRR

**Figure 3:**
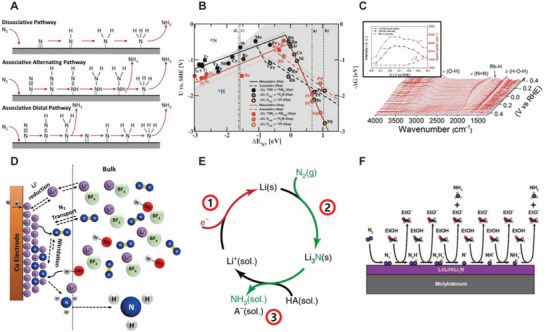
Reaction pathways of DNRR and INRR A) Scheme for three possible DNRR mechanisms: dissociative, associative alternating, and associative distal pathways. Reproduced with permission.^[^ ^18^ ^]^ Copyright 2016, Elsevier. B) Volcano plot for DNRR on metal electrodes depending on the mechanistic pathways. The volcano plot for the HER is overlaid for comparison. Reproduced with permission.^[^ ^26^ ^]^ Copyright 2012, RSC. C) SEIRAS spectra of an Rh film electrode in N_2_‐saturated 0.1 m KOH. Reproduced with permission.^[^ ^3^ ^]^ Copyright 2020, Wiley VCH. D) Overall reaction schemes of Li‐mediated electrochemical nitrogen reduction on Cu electrode. Reproduced with permission.^[^ ^78^ ^]^ Copyright 2019, Elsevier. E) Stepwise Li‐mediated catalytic cycle for nitrogen reduction to ammonia. Reproduced with permission.^[^ ^76^ ^]^ Copyright 2020, Springer Nature. F) Heterogeneous nitrogen reduction on lithium metal surface, which is similar to DNRR pathway. Lithium (or LiH, Li_3_N) layers are not involved in the cycle, instead, provide the binding sites to N_2_ and nitrogen‐containing adsorbates. Reproduced with permission.^[^ ^61^ ^]^ Copyright 2020, Wiley VCH.

Three elementary steps are typically considered for DNRR (**Figure** **3**A): 1) chemical adsorption of N_2_ molecules and protons onto the cathode surface, 2) activation of N_2_ and hydrogenation, and 3) rearrangement and desorption of the product NH_3_ or other products (e.g., hydrazine [N_2_H_4_] and diazene [N_2_H_2_]) from the electrode surface and their migration into the electrolyte.^[^ ^10^, ^26^, ^57^, ^58^ ^]^ The Haber–Bosch process produces NH_3_ via a dissociation pathway because of high pressure and temperature operating conditions. In contrast, biological systems adopt an associative pathway to generate NH_3_, which involves N_2_ fixation via the enzymatic process of nitrogenases.^[^ ^31^, ^59^ ^]^ For electrochemical nitrogen reduction, an associative path would be more appropriate because ambient reaction conditions are used in electrochemical systems, necessitating nitrogen adsorption and protonation as the first step for an efficient eNRR.^[^ ^60^ ^]^ The associative pathway can be further categorized into alternating and distal pathways based on the sequence of hydrogenation on the nitrogen atoms. Specifically, in the associative alternating pathway, hydrogenation occurs alternatively on the two nitrogen atoms, while in the associative distal pathway, it occurs on one nitrogen atom and then the other.

Because N_2_ adsorption/activation is consistent with the formation of N_2_H* species on the electrode, Medford et al. proposed that Brønsted–Evans–Polanyi (BEP) relationships result in a volcano plot, which helps to visualize the ability of metals to reduce N_2_ electrochemically (Figure 3B).^[^ ^25^, ^26^, ^98^ ^]^ The model focuses on the eNRR rate‐determining step (RDS) on catalyst materials, depending on the eNRR pathways (solid lines for the dissociative pathway and dotted lines for the associative pathway) and the surface atomic geometry (flat surfaces for black and stepped surfaces for red). For metals on the left side of the volcano plot, the hydrogenation process is considered the RDS in both the associative and dissociative pathways (*NH_2_ + H^+^ + e^−^ → NH_3_ on the stepped surfaces and *NH + H^+^ + e^−^ → *NH_2_ for the flat surfaces). However, for metals on the right side of the plot, the RDSs are changed depending on the reaction pathways; the first protonation step of N_2_ (N_2_ → *N_2_H) for the associative pathway and the N_2_ splitting step (N_2_ → *2N) for the dissociative pathway regardless of the surface atomic geometries. It is concluded that the flat surfaces of transition metals such as Sc, Y, Ti, and Zr effectively reduce N_2_ to NH_3_ through the dissociative mechanism with the applied potential of −1 *V*_NHE_ to −1.5 *V*_NHE_, by comparing the free energies of *N (white area), *H (gray‐shaded area), and intermediates (those species are stable on the left side area from the vertical lines a) *NH, b) *NH_2_, c) *N_2_H, and d) *N_2_H_2_) on the catalyst surfaces. Furthermore, the slow rate of N_2_ reduction observed on catalysts such as Pt, Pd, or Ru can be explained by free energy calculations. These calculations suggest that, under negative bias, these catalyst materials become saturated with hydrogen, hindering the adsorption of nitrogen and subsequently impeding NH_3_ production. Also, N vacancy incorporated Mars‐van Krevelen (MvK) mechanism has been suggested by Yang et al. In the MvK mechanism, a lattice N on the surface of transition metal nitride catalysts is consumed to produce NH_3,_ leaving an N vacancy, which is reoccupied by adsorbed N_2_.^[^ ^62^ ^]^ Despite the efforts to reveal the nature of DNRR process, eNRR mechanism, however, has remained elusive.^[^ ^63^, ^64^, ^65^, ^66^ ^]^

#### 2.1.2 Spectroscopic Studies

Due to the low yields of NH_3_ from DNRR, it is challenging to study the reaction intermediate species. A few reports have investigated the reaction intermediates using surface‐enhanced infrared spectroscopy (SEIRAS) and differential electrochemical mass spectrometry (DEMS) (Figure 3C).^[^ ^67^, ^68^, ^69^ ^]^ Using the SEIRAS, Yao et al. discovered the potential‐dependent molecular characteristics on the Rh electrode surface. Figure 3C inset plots the band center (cm^−1^) and intensity of each stretching mode of N = N & Rh‐H species versus applied voltages. The authors revealed that the extent of coverages of the N_2_H*_x_* and H species on the Rh electrode strongly depends on the applied potentials. Notably, maximum N_2_H*_x_* coverage was obtained between 0 and −0.2 V, and H deposition on the electrode surface increased below −0.2 V (inset of Figure 3C). Also, by integrating SEIRAS and DEMS, it was discovered that the final protonation of *N_2_H_2_ to NH_3_ on the Rh electrodes is the RDS, which is consistent with the theoretical expectation for the associative eNRR mechanism, as suggested by Medford et al. N_2_H*_x_* (0 ≥ *x* ≥ 2) was detected using SEIRAS and DEMS, with an N═N stretching mode at ≈2020 cm^−1^ and a signal at *m/z* = 29 (N_2_H^+^), respectively. However, the N_2_H_2_^+^ species was not detected at *m/z* = 30. The same group also determined the DNRR mechanisms on the Ru, Au, and Pt electrode surfaces, but the N_2_H_2_^+^ species have not been detected thus far. These studies suggest an alternative mechanism via the facile formation of N_2_H*_x_* species on the catalysts and decomposition in the electrolytes.

### 2.2 INRR (Li‐Mediated eNRR)

#### 2.2.1 Development of Li‐Mediated Nitrogen Reduction Methodology

The Li‐mediated eNRR started back in the 1990s. After it was first suggested by Fichter et al. in 1931,^[^ ^70^ ^]^ Tsuneto et al. reported Li‐mediated electrochemical N_2_ fixation via organic solutions containing a small amount of proton source as an electrolyte to suppress the competing reaction, HER.^[^ ^71^, ^72^ ^]^ The experiments were performed under 50 atm N_2_ pressure with LiClO_4_ (0.2 m) and ethanol (0.18 m) in a tetrahydrofuran (THF) electrolyte. The selectivity toward NH_3_ was low, and it required a high nitrogen pressure.

McEnaney et al. proposed a strategy to separate N_2_ reduction from the subsequent protonation to NH_3_ in their molten‐salt system.^[^ ^73^ ^]^ The suggested phase diagram of Li─N─O─H incorporates the following Li cycle steps: 1) Reductive LiOH electrolysis generates Li in the hydrogen‐free environment. The phase diagram indicates that Li exists as a stable phase above −3.3 V versus the standard hydrogen electrode (SHE). 2) Introduction of 1 bar of nitrogen gas into the activated Li surface facilitates Li nitridation. Lithium nitride (Li_3_N) is demonstrated to be more stable than Li for all potentials, implying that Li_3_N formation is energetically favorable under mild conditions. 3) Li_3_N is rapidly hydrolyzed to NH_3_ upon adding water at 0 V versus SHE. Li_3_N decomposes to stable LiOH and NH_3_. Although McEnaney et al. successfully synthesized NH_3_ with 88.5% FE at low N_2_ pressure, their system had several practical limitations; for example, high operating conditions (> 400 °C) were required for providing a continuous liquid phase of molten salt electrolyte, which inevitably resulted in the formation of liquid Li metal (melting point = 180.5 °C). Thus, additional steps were necessary to separate liquid Li. Kwiyong et al. reported an alternative strategy using a Li‐ion conducting glass ceramic material (LISICON) system. Although elevated temperature (220 °C) and stepwise processes from different reaction chambers are required, LISICON allows Li^+^ ion migration and Li deposition at room temperature.^[^ ^74^ ^]^ Recently, several studies have been conducted on continuous Li‐mediated nitrogen reduction catalysis under mild conditions without any separation steps.^[^ ^75^, ^76^, ^77^ ^]^ Protons from external proton donors or anodic reactions are fed into the catholyte, which directly reacts with the Li_3_N layers to yield NH_3_. This approach has received significant attention as it is a one‐pot synthesis without harsh operating conditions.

#### 2.2.2 Proposed Mechanisms for INRR

The overall reaction of Li‐mediated electrochemical nitrogen reduction on the electrode is depicted in Figure 3D. This diagram well illustrates the key components of the reaction.^[^ ^78^ ^]^ The mechanism of continuous Li‐mediated nitrogen reduction typically involves three steps (Figure 3E): 1) a highly reactive Li layer is formed on the electrode surface, 2) Li_3_N is formed when Li reacts with dissolved N_2_, and 3) NH_3_ is produced via sequential protonation, resulting in Li^+^ ions in the electrolyte that close the catalytic cycle. Based on this concept, the Li redox couple is “mediated” to complete mixed electrolytic and chemical reactions. The detailed mechanism can be subcategorized depending on whether each pathway is concerted or proceeds stepwise.

Cai et al. experimentally demonstrated three stepwise mechanisms, as suggested in Figure 3E.^[^ ^79^ ^]^ They observed that NH_3_ continued to be produced from N_2_ even when the voltage was no longer applied after a certain period of electrolysis. They claimed that the Li metal layers on the electrode remained sufficiently reactive to continue the N_2_ splitting cycle until Li was completely consumed. Several control experiments were performed to test this hypothesis. They found that the reductive current values remained almost unchanged under N_2_ and Ar environments or with and without ethanol. This implies that N_2_ reduction or ethanol splitting is not involved in the electrochemical process, and the injected electrons are only consumed for Li plating.

For the concerted pathway of (1) and (3), the expulsion of NH_3_ from Li_3_N and Li redeposition was suggested. Andersen et al. suggested kinetic models wherein electrochemical Li deposition is accompanied by hydrogenation of the adsorbed N_2_ molecules.^[^ ^80^ ^]^ Based on this mechanism, they reported that high‐energy electrons from highly negative potentials could be restored in the Li layer in the absence of an electric field and could be used to produce NH_3_ from N_2_. Additionally, Ma et al. suggested a (1) and (2) concerted model for N_2_ fixation in the Li–N_2_ battery cycle.^[^ ^81^ ^]^ During the discharging step, Li^+^ reacts with nitrogen to form Li_3_N via an electrochemical process, while the Li anode provides Li^+^ ions.

To elucidate the cycle with a concerted proton‐electron transfer model, Schwalbe et al. hypothesized that the long‐lived Li metal layer might provide heterogeneous active sites, which assist the coupled proton–electron transfer in the catalytic cycle.^[^ ^61^ ^]^ Figure 3F depicts the stepwise reaction pathways to yield NH_3_ from N_2_, analogous to the direct eNRR pathway. In this mechanism, Li^+^ is no longer participating in the catalytic cycle. Instead, N_2_ splitting and its hydrogenation become voltage‐dependent processes on the Li metal layer. The authors suggested that the mixed Li*_x_* N*_y_* H*_z_* species (e.g., lithium imide or lithium amide) formed on the electrode surface can act as catalytically active intermediates for the eNRR.

#### 2.2.3 Electrokinetic Studies

**Figure 4:**
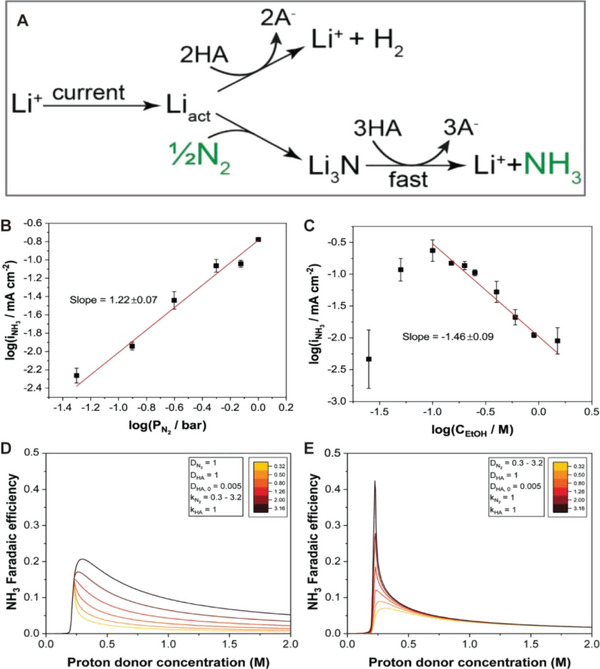
Electrokinetic studies of INRR. A) Two reaction pathways occur in the Li‐mediated NRR. When the lithium layer is formed by reduction, it is spontaneously consumed through either hydrogen evolution (upper) or ammonia formation (bottom) with the help of proton donor. Log–log plot of partial current density toward NH_3_ with varying B) partial pressures of N_2_ and C) ethanol concentration as proton donor. Reproduced with permission.^[^ ^75^ ^]^ Copyright 2019, Elsevier. Simulated Faradaic efficiency toward NH_3_ depending on the proton donor concentration at different D) rate constant ($k_{N_{2}})$ for nitrogen reduction and E) diffusivity of nitrogen ($D_{N_{2}})$. Reproduced with permission.^[^ ^77^ ^]^ Copyright 2022, American Chemical Society.

Several research groups have endeavored to elucidate the complex kinetic nature of Li‐mediated nitrogen reduction.^[^ ^82^, ^83^ ^]^ Lazouski et al. reported continuous Li‐NRR (0.2 m LiClO_4_ and 0.2 m ethanol in THF) under ambient conditions. Furthermore, they attempted to analyze the overall kinetics of NRR through a series of electrokinetic studies.^[^ ^75^ ^]^ **Figure** **4**A depicts two possible reaction routes in the Li‐NRR. After being plated on the electrode, Li can react with either N_2_ to generate Li_3_N or with a proton source (HA) to produce molecular hydrogen. Li_3_N yields NH_3_ by reacting with HA. The rate of each pathway can be expressed as follows

(3) $$r_{H_{2}}=k_{1}{\left[\mathrm{Li}\right]}^{\alpha }{\left[\mathrm{EtOH}\right]}^{x}$$

(4) $$r_{{\mathrm{NH}}_{3}}=k_{2}{\left[\mathrm{Li}\right]}^{\beta }{\left[N_{2}\right]}^{1}$$

The reaction order for nitrogen pressure was found to be first‐order (Figure 4B) from the dependence of the partial current density toward NH_3_ ($i_{{\mathrm{NH}}_{3}}$) on the nitrogen partial pressure (Equation (2)). Similarly, Figure 4C displays the log–log plot of the $i_{{\mathrm{NH}}_{3}}$ versus the concentration of ethanol, a proton donor. The NH_3_ partial current first increases with ethanol concentration up to 0.1 m, and then it monotonically decreases, exhibiting a −1.5 order dependence on ethanol concentrations. The authors claimed that the obtained negative reaction order was attributed to the contribution from the hydrogen evolution (Figure 4A, top), which depleted the available Li layer for nitridation.

The subsequent hydrogenation of Li_3_N to NH_3_ was assumed to be a rapid step because the Li_3_N layer was scarcely observed in the cathode. Li_3_N formation was considered the RDS. Because the partial current toward hydrogen was considerably higher than that toward NH_3_, the authors further assumed that 1) the reaction rate should be $r_{{\mathrm{NH}}_{3}}\ll r_{H_{2}}$, 2) Li was in a quasisteady state during NRR (the rate of change in Li concentration was ≈0), and 3) most of the applied current was used to generate the Li layer. The abovementioned experimental results and assumptions lead to the following relationships

(5) $$\begin{array}{ccc} F\frac{d\left[\mathrm{Li}\right]}{dt} & = & I-k_{1}{\left[\mathrm{Li}\right]}^{\alpha }{\left[\mathrm{EtOH}\right]}^{x}-k_{2}{\left[\mathrm{Li}\right]}^{\beta }{\left[N_{2}\right]}^{1}\\ & \approx & I-k_{1}{\left[\mathrm{Li}\right]}^{\alpha }{\left[\mathrm{EtOH}\right]}^{x}\approx 0 \end{array}$$

(6) $$\left[\mathrm{Li}\right]={\left(\frac{I}{k_{1}{\left[\mathrm{EtOH}\right]}^{x}}\right)}^{\frac{1}{\alpha }}$$

Subsequently, the obtained Li concentration (Equation (4)) was employed to correlate $FE_{{\mathrm{NH}}_{3}}$ and total current. $FE_{{\mathrm{NH}}_{3}}$ showed a one‐half‐order dependence on the total current, resulting in the following expression, which explains the dependence of $FE_{{\mathrm{NH}}_{3}}$ on the reacting species and current

(7) $$\begin{array}{ccc} FE_{{\mathrm{NH}}_{3}} & = & \frac{k_{2}{\left[\mathrm{Li}\right]}^{\beta }{\left[N_{2}\right]}^{1}}{I}\approx \frac{k_{2}{\left[\mathrm{Li}\right]}^{\beta }{\left[N_{2}\right]}^{1}}{k_{1}{\left[\mathrm{Li}\right]}^{\alpha }{\left[\mathrm{EtOH}\right]}^{x}}\\ & = & \left(\frac{k_{2}}{k^{1+\frac{\beta -\alpha }{\alpha }}}\right)\frac{\left[N_{2}\right]}{{\left[\mathrm{EtOH}\right]}^{x\left(1+\frac{\beta -\alpha }{\alpha }\right)}}I^{\frac{\beta -\alpha }{\alpha }} \end{array}$$

(8) $$\frac{\beta -\alpha }{\alpha }=\frac{1}{2},\;-x\left(1+\frac{\beta -\alpha }{\alpha }\right)=-x\left(1+\frac{1}{2}\right)=-1.5$$

The ethanol reaction order *x* was deduced to be unity and the Li reaction order, *α*, and *β*, to 2 and 3, respectively.

In the subsequent study, Lazouski et al. further endeavored to describe the role of proton donors in terms of transport kinetics. They attempted to correlate the permeability of the solid electrolyte interface (SEI) and the types/concentrations of proton donors.^[^ ^77^ ^]^ They postulated that two different types of SEIs (permeable and impermeable) coexist in the system; the permeable SEI fraction was denoted by *θ*. The effective diffusivity of 1) nitrogen and 2) proton donors through the SEI was considered as a critical parameter of *θ* in their model

(9) $$N_{j}=\left(D_{j,i}\left(1-\theta \right)+D_{j,p}\theta \right)\left(C_{j,\mathrm{bulk}}-C_{j,s}\right)$$

Where *D_j_* is the diffusivity and *N_j_* is the total diffusion flux of species *j* through the SEI; *C_j_*_,bulk_ and *C_j,s_* are the concentrations of species *j* at the SEI and at the reactive electrode surface beneath the SEI, respectively. At a steady state, the rate of the NRR should be equal to that of nitrogen diffusion through the SEI

(10) $$\begin{array}{ccc} r_{N_{2}} & = & k_{N_{2}}C_{N_{2},S}C_{\mathrm{Li}}=\left(D_{N_{2},i}\left(1-\theta \right)+D_{N_{2},p}\theta \right)\left(C_{N_{2},\mathrm{bulk}}-C_{N_{2},s}\right)\\ & = & N_{N_{2}} \end{array}$$

The rate of all the reactions involving the proton donor can be expressed as follows. *r*_HA,red_ indicates the reduction rate toward hydrogen evolution, and *r*_HA,prot_ is protonation rate of dinitrogen molecule to ammonia

(11) $$r_{\mathrm{HA},\mathrm{red}}=k_{\mathrm{HA}}C_{\mathrm{HA},S}C_{\mathrm{Li}}$$

(12) $$r_{\mathrm{HA},\mathrm{prot}}=6k_{N_{2}}C_{N_{2},S}C_{\mathrm{Li}}$$

(13) $$\begin{array}{ccc} r_{\mathrm{HA}} & = & k_{\mathrm{HA}}C_{\mathrm{HA},S}C_{\mathrm{Li}}+6k_{N_{2}}C_{N_{2},S}C_{\mathrm{Li}}\\ & = & \left(D_{\mathrm{HA},i}\left(1-\theta \right)+D_{\mathrm{HA},p}\theta \right)\left(C_{\mathrm{HA},\mathrm{bulk}}-C_{\mathrm{HA},S}\right)=N_{\mathrm{HA}} \end{array}$$

Similar to Equation (3), the accumulation rate of the Li layer can be expressed using the current density and consumption rate (Equation (12))

(14) $$F\frac{dC_{\mathrm{Li}}}{dt}=I-F\left(k_{\mathrm{HA}}C_{\mathrm{HA},S}C_{\mathrm{Li}}+6k_{N_{2}}C_{N_{2},S}C_{\mathrm{Li}}\right)\geq 0$$

The surface concentrations of N_2_, HA, and Li species $\left(C_{N_{2},S},\;C_{\mathrm{HA},S}\;C_{\mathrm{Li}}\right)$ can be obtained from Equation (12) as assuming steady‐state conditions and constraints from Equations (8) and (9). Considering the stoichiometry of the reacting species and the relationship between FE and the reaction rate, the following equations are derived

(15) $$r_{{\mathrm{NH}}_{3}}=2\cdot \min\left(r_{N_{2}}\right)$$

(16) $$FE_{{\mathrm{NH}}_{3}}=\frac{3F\;r_{{\mathrm{NH}}_{3}}}{I}$$

The rate of NH_3_ evolution is limited by the diffusivity of nitrogen (Equation (8)). The FE for hydrogen is then derived by subtracting the NH_3_ rate from the total HA‐involved reaction rate

(17) $$FE_{H_{2}}=\frac{F\cdot (r_{\mathrm{HA}}-3r_{{\mathrm{NH}}_{3}})}{I}$$

Therefore, to minimize the $FE_{H_{2}}$, the proton diffusion rate should be controlled to limit NH_3_ formation (Equation (15)). Based on the kinetic diffusion model, the authors attempted to reveal the effects of diffusivity and rate constants. Figure 4D,E shows that the peak $FE_{{\mathrm{NH}}_{3}}$ is expected to be more strongly affected by the relative diffusivities of N_2_ and H_2_ than by the overall rate constant for the NRR. Because commonly used proton donors are sterically bulky, the nitrogen diffusivity in the SEI layer is typically higher than that of the proton donor. Thus, even if the rate constant for N_2_ reduction is lower than for proton donor reduction, a high FE can still be achieved as diffusion of nitrogen is favored over proton in SEI. This implies that the SEI serves as a protective layer for selective NH_3_ production by suppressing undesirable hydrogen evolution and exclusively promoting N_2_ transport.

## 3 Strategies to Advance the eNRR Performance

### 3.1 Catalyst Design Strategies for Enhancing DNRR Performances

The practical applications of the eNRR are hindered by the slow reaction kinetics and competitive HER, resulting in low FE. N_2_ is composed of two N atoms, each with five valence electrons in the 2s and 2p orbitals. The electrons occupy all bonding orbitals and one σ* orbital, making this chemically stable form. In terms of charge exchange, donating electrons from an occupied N_2_ *σ* orbital to an empty metal *d* orbital, with the back donation from an occupied metal *d* orbital to an empty N_2_ π* orbital, weakens the triple N─N bonds and promotes eNRR.^[^ ^84^ ^]^ Therefore, the electrocatalyst design can efficiently enhance eNRR performance because the charge exchange property is directly related to the chemical features of the catalyst surface.^[^ ^3^, ^85^ ^]^ Direct electrochemical NH_3_ synthesis from nitrogen and water is a simple alternative to the conventional Haber–Bosch method.^[^ ^3^ ^]^ Carbon‐based materials, metals, nonmetals, metal oxides, metal sulfides, metal nitrides, bimetallic and ternary nitrides, and single‐atom catalysts have been developed as catalysts for the eNRR in aqueous electrolytes. The intrinsic activity of the active sites is influenced by the electronic structure of the catalyst, which can be regulated through defect/strain engineering.^[^ ^85^ ^]^ In contrast, the apparent activity, which depends on the yield, is influenced by the structure and morphology of the catalyst. The apparent activity of the electrocatalyst can be enhanced through surface area modification, pore engineering, and hybridization. Herein, we discuss the effects of defect engineering, surface geometry/morphology, and heterostructure formation on the eNRR performance of electrocatalysts in aqueous electrolytes.

#### 3.1.1 Defect Engineering

**Figure 5:**
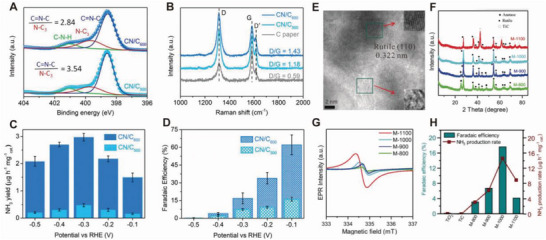
Defect engineering of electrocatalysts. A) N 1s XPS spectra of CN/C_500_ and CN/C_600_, B) Raman spectra of CN/C_500_, CN/C_600_, and C paper, C) NH_3_ yield rate, and D) Faradaic efficiency of CN/C_600_ and CN/C_500_ at different potentials. Reproduced with permission.^[^ ^87^ ^]^ Copyright 2020, American Chemical Society. E) TEM image, F) XRD pattern, G) EPR spectrum, and H) FE and NH_3_ production rate of various C–Ti*_x_* O*_y_*/C hybrids. Reproduced with permission.^[^ ^89^ ^]^ Copyright 2019, Wiley.

Defect engineering is an efficient strategy for regulating the electronic structure of electrocatalysts and consequently enhancing N_2_ adsorption.^[^ ^86^, ^87^ ^]^ Moreover, defect engineering alters the adsorption energy of the intermediates, thereby suppressing the HER and improving the selectivity of eNRR. The defects can primarily be induced by doping with heteroatoms or by forming vacancies. Doping induces charge redistribution and improves the charge transfer abilities of the electrocatalyst.^[^ ^88^ ^]^ Boron, nitrogen, and sulfur are commonly used as dopants in electrocatalysis. When graphene is doped with 6.2% boron, the NH_3_ yield rate and FE of the doped graphene increase by 5 and 10 times, respectively, compared with those of the undoped graphene.^[^ ^86^ ^]^ This increase in performance is attributed to the difference in electronegativity between boron (2.04) and carbon (2.55). The electron‐deficient boron sites have an increased tendency to bind to N_2_, thus enabling the formation of B─N bonds and the subsequent production of NH_3_. Similarly, Wang et al. reported the role of N‐doping in improving the eNRR activity of NiO nanosheet arrays on carbon cloth (NiO/CC).^[^ ^88^ ^]^ Initially, a hydrothermal method was used to synthesize NiO/CC, which was subsequently doped with nitrogen (N–NiO/CC) by annealing in an Ar/NH_3_ atmosphere. As deduced from density function theory (DFT) computations, N doping modified the electronic structure of NiO, thereby promoting the activation of N_2_, improving the stabilization of the key intermediate *NNH, and decreasing the reaction energy barrier. Consequently, the eNRR activity was enhanced. N─NiO/CC resulted in an NH_3_ production rate of 22.7 µg h^−1^ mg^−1^ and 7.3% FE in 0.1 m LiClO_4_ aqueous electrolyte at −0.5 V versus the reversible hydrogen electrode (RHE). In contrast, vacancies create active sites with abundant localized electrons, which can weaken N≡N.^[^ ^89^ ^]^ Peng et al. reported the eNRR activity of carbon nitride with N_2C_ vacancies, which was grown on carbon fiber paper (CN/C) through chemical vapor‐assisted synthesis.^[^ ^90^ ^]^ CN with perfect planes is characterized by low catalytic activity, whereas CN with N_2C_ defects exhibits strong interactions with N_2_. The growth conditions can control the number of vacancies on CN. CN/C_600_ synthesized at 600 °C has more catalytic sites and a higher electrochemically active surface area (ECSA) than CN/C_500_. The ratio of deconvoluted peaks (C═N─C/N─C_3_) in the N 1s spectra of CN/C_600_ and CN/C_500_ (**Figure** **5**A) indicates the presence of C═N─C N_2C_ vacancies in CN/C_600_. In addition, the increased ratios of D/G peaks in the Raman spectrum of CN/C_600_ (Figure 5B) indicate an increased number of defects compared with those in CN/C_500_ and bare carbon paper. Based on the slope of the m_NH3_–time plot, the NH_3_ yield and FE for CN/C_600_ at −0.3 V versus RHE were evaluated as 2.9 µg mg_cat_^−1^ h^−1^ and 16.8% FE, respectively (5.7 times higher than those for CN/C_500_) (Figure 5C,D).

Jin et al. reported the eNRR activity of W_2_N_3_ nanosheets with stable nitrogen vacancies.^[^ ^91^ ^]^ Long‐term electrolysis and postcharacterization techniques indicated the stability of nitrogen vacancies in the W_2_N_3_ nanosheets. DFT calculations predicted that the high stability of these vacancies could be attributed to the 2D confinement effect and the high valence state of the W atoms. Furthermore, active catalytic centers could be created by incorporating anions into metal oxides, thereby improving the eNRR performance.^[^ ^92^ ^]^ C─Ti*_x_* O*_y_*/C structures were obtained by annealing MIL‐125 (Ti) (a Ti‐based metal–organic framework) at different temperatures (800—1100 °C). The oxygen vacancies in TiO_2_ were substituted with carbon atoms, thus forming Ti─C bonds in C─Ti*_x_* O*_y_*. The high‐resolution transmission electron microscopy (TEM) image of M‐1000 (MIL‐125 (Ti) annealed at 1000 °C) indicates the uniform dispersion of TiO_2_ nanoparticles (NPs) ranging between 3 and 20 nm in an amorphous environment (Figure 5E). In the X‐ray diffraction (XRD) pattern (Figure 5F), peaks attributed to tetragonal rutile or anatase are observed. However, as the temperature increases from 800 to 1100 °C, peaks attributed to TiC are more dominant than rutile or anatase titania. The increased intensity of the peaks in the electron paramagnetic resonance (EPR) spectrum indicates a higher concentration of oxygen vacancies at higher temperatures (Figure 5G). The eNRR performance of C─Ti*_x_* O*_y_*/C is improved, with an NH_3_ yield rate of 14.8 mg h^−1^ mg_cat_^−1^ and 17.8% FE (Figure 5H).

#### 3.1.2 Surface Geometry/Morphology Engineering

The apparent activity of the electrocatalyst can also be enhanced by tailoring the morphology of nanostructures and engineering the surface chemistry.^[^ ^93^, ^94^, ^95^ ^]^ 3D bimetallic PdRu porous nanomaterials with interconnected voids and skeletons exhibit promising eNRR performance.^[^ ^93^ ^]^ These porous electrocatalysts provide numerous active sites for reducing N_2_ to NH_3_ and facilitate the mass transfer of N_2_. Porous bimetallic PdRu provides a satisfactory NH_3_ yield, which declines by only 8% after long‐term stability tests. Also, engineering the surface chemistry through appropriate electrolyte is important for enhancing eNRR performances. Wang et al. prepared a porous Au film electrodeposited on Ni foam (Au/NF) through micelle‐assisted deposition, and the Au/NF exhibited excellent eNRR performance. Compared to the total current density at −0.2 *V*_RHE_ in 0.1 m KOH, a lower total current density was obtained in 0.1 m Na_2_SO_4_ (≈0.25 mA cm^−2^ in 0.1 m Na_2_SO_4_ and ≈3.3 mA cm^−2^ in 0.1 m KOH), indicating the suppression of the HER in 0.1 m Na_2_SO_4_ due to the low charge and mass transfers. However, similar NH_3_ yield rates were obtained in both electrolytes (9.42 µg h^−1^ cm^−2^ in 0.1 m Na_2_SO_4_ and 10.50 µg h^−1^ cm^−2^ in 0.1 m KOH), and the calculated FE was 9 times higher in 0.1 m Na_2_SO_4_ than in 0.1 m KOH (13.36% in 0.1 m Na_2_SO_4_ and 1.42% in 0.1 m KOH). In this case, Na_2_SO_4_ can be considered a promising electrolyte for the eNRR on the porous binder‐free Au/NF electrode.^[^ ^96^ ^]^

**Figure 6:**
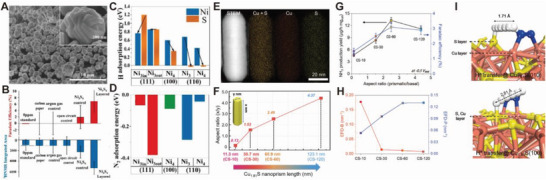
Engineering the surface/morphology of the electrocatalysts. SEM images of A) Ni_3_S_4_ powder with flat facets and the control Ni_3_S_4_ powder with irregular surfaces. B) Comparison of FEs for samples and blank controls used for subtracting the spurious signal. C) H adsorption energies on all facets referenced to 1/2H_2_. D) N_2_ adsorption energy for all Ni sites. Reproduced with permission.^[^ ^94^ ^]^ Copyright 2021, American Chemical Society. E) HAADF‐STEM image and corresponding elemental mapping analysis of Cu_1.81_S hexagonal nanoprism. F) aspect ratio of the nanoprisms. G) NH_3_ production yield and Faradaic efficiency. H) exposed facet densities (nm^−1^) at basal (EFD‐B; red) and prismatic (EFD‐P; blue) planes. I) simulations of H* transfer configurations on Cu_1.81_S(010) and Cu_1.81_S(100). Reproduced with permission.^[^ ^99^ ^]^ Copyright 2022, American Chemical Society.

Engineering surface features on an atomic scale has shown several insightful results for improving eNRR performance. For example, MoS_2_ nanoflowers with disordered atomic arrangements and crystal defects are regarded as efficient catalysts for the eNRR.^[^ ^97^ ^]^ The disorder and defects improve the stability of the nanoflower by tuning the properties and activity of the reactive sites. Irregularly structured catalysts with high‐index facets and multiple edge/corner sites are favored because of their effective binding with the reactants.^[^ ^98^ ^]^ In contrast, Hao et al. showed that the eNRR performance of a Ni_3_S_4_ catalyst with low energy and a smooth surface was higher than that of those with distorted surfaces.^[^ ^94^ ^]^ Ni_3_S_4_ prepared using two different synthetic protocols resulted in extended flat facets and irregular surfaces, as shown in the scanning electron microscopy (SEM) images in **Figure** **6**A (right image for the flat facet and left inset image for the irregular surface in the insets). The eNRR reaction products collected after 2 h were quantified using ^1^H‐NMR (^1^H nuclear magnetic resonance) spectroscopy under extended scan times, and spurious signals were eliminated based on control experiments under varied synthesis conditions. The catalytic activity of low‐energy Ni_3_S_4_ with flat facets was three times higher than that of Ni_3_S_4_ with a distorted surface (Figure 6B). An NH_3_ production rate of 1.28 µg mg^−1^ h^−1^ and 6.8 ± 3.3% FE were achieved for Ni_3_S_4_ with smooth facets, whereas the FE of the distorted Ni_3_S_4_ was only 1.9 ± 3.6%. According to DFT calculations, the availability of multiple vacant Ni sites and the competing nitrogen and hydrogen adsorption influence the enhanced eNRR activity of the smooth facets (Figure 6C,D). Coordinatively vacant Ni sites in the flat low‐energy facets are readily available for N_2_ binding, thus improving the eNRR performance.

Accordingly, Jin et al. emphasized the importance of the coordination environment in catalytic activity and reported the influence of the atomic geometry of NPs on the eNRR kinetics.^[^ ^99^ ^]^ The dependence of eNRR activity on the surface geometry of copper sulfide (Cu_1.81_S) nanocatalysts with exposed facets of (100) and (010) for smooth and zigzag planes, respectively, was investigated. High‐angle annular dark‐field scanning transmission electron microscopy (HAADF‐STEM) images indicate the uniform distribution of Cu and S with the aspect ratio differences from 0.13 for CS‐10 to 4.37 for CS‐120 (x and y indicate zigzag and smooth planes, respectively) (Figure 6E,F). The NH_3_ production rate and FE increase with the increasing length of the hexagonal nanoprisms (Figure 6G); the improvement in the eNRR performance is associated with the prismatic plane density (Figure 6H). Also, as the prismatic plane density increased, lower current density was obtained at −0.5 *V*_RHE_ under Ar saturated electrolyte. This result indicates that the zigzag surface geometry limits the competitive HER, which is consistent with the results of eNRR. DFT calculations reveal that the protruded atoms on the zigzag planes of Cu_1.81_S nanoprisms have a reduced proton transfer distance from 2.81 Å on the (100) to 1.71 Å on the (010), and H efficiently migrates from H*@S to N_2_*@Cu, thus improving the eNRR kinetics (Figure 6I).

The catalytic performance of single‐atom catalysts can be improved by engineering their geometry through metal atoms or coordination anions.^[^ ^100^, ^101^ ^]^ For example, a tungsten single‐atom catalyst with oxygen and nitrogen coordination and a metal loading exceeding 10 wt% provides satisfactory eNRR performance.^[^ ^101^ ^]^ The O and N coordination on tungsten influences its adsorption behavior and selectivity, thus improving the performance. Li et al. reported a strategy of interfacial polarization to facilitate the breaking of N≡N bonds and enhance NH_3_ production.^[^ ^102^ ^]^ The electric fields produced from the protruding Fe single‐atom catalysts immobilized on MoS_2_ can polarize N_2_ by attenuating the interfacial energy barrier. The resultant polarization fields between N_2_ and Fe–MoS_2_ drive additional electrons into the antibonding orbitals of N_2_, thus improving the NH_3_ production rate. Therefore, the morphology of the nanomaterial affects the electric field that is generated to polarize N≡N.

#### 3.1.3 Heterostructure

Heterostructure engineering is a facile strategy to enhance the catalytic activity of nanomaterials. Owing to the synergy from individual components in multicomponent nanostructures (hybrid structures), their eNRR activity is higher than that of the individual components.^[^ ^103^, ^104^ ^]^ The interface between two components in hybrid structures imparts unique properties to the individual components, promoting abundant electron channels and accelerating the electron transfer rate.^[^ ^105^ ^]^ Interface engineering primarily includes metal (derivative)–carbon, metal (derivative)–metal oxide, and intermetallic interfaces.^[^ ^106^ ^]^ SnO_2_ quantum dots (QDs) distributed on reduced graphene oxide (SnO_2_/RGO), synthesized via self‐propagating combustion, are demonstrated to be efficient catalysts for producing NH_3_ at a rate of 25.6 µg h^−1^ mg^−1^ and 7.1% FE in 0.1 m Na_2_SO_4_ at −0.5 V (vs RHE).^[^ ^107^ ^]^ The RGO support improves the conductivity of the SnO_2_ QDs and prevents their agglomeration, thereby increasing the surface area and number of active sites for N_2_ adsorption. DFT calculations reveal that the highly conductive hybrid structure lowers the energy barrier of *N_2_ → *N_2_H and promotes eNRR activation. Wang et al. observed a similar phenomenon in a CuO/graphene nanocomposite.^[^ ^104^ ^]^ The interface engineering of 2D/2D materials enables face‐to‐face contact, facilitating strong electronic interactions at the interface.^[^ ^108^ ^]^ In the MoS_2_/C_3_N_4_ heterostructure, electrons migrate from C_3_N_4_ to MoS_2_ across the interface, thus creating a charge density difference. This electron redistribution significantly increases the conductivity of MoS_2_/C_3_N_4_. The crucial intermediate *N_2_H is stabilized on the Mo edge sites of MoS_2,_ and the reaction energy barrier decreases. Furthermore, *H is adsorbed on the S edge sites, thus protecting the NRR‐active Mo edge sites from the competing HER.

**Figure 7:**
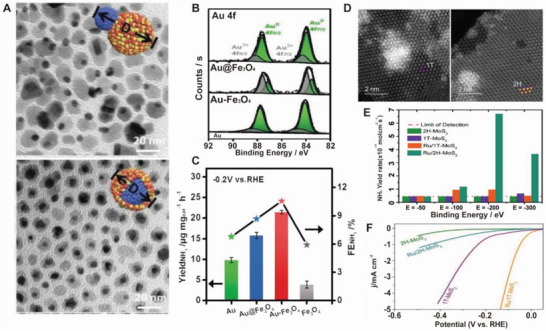
Heterostructure engineering of electrocatalysts. A) TEM images of Au–Fe_3_O_4_ (up) and Au@Fe_3_O_4_ (down) nanoparticles. B) XPS curves of Au 4f for different catalysts. C) yield and Faraday efficiency of different catalysts at −0.2 V versus RHE. Reproduced with permission.^[^ ^103^ ^]^ Copyright 2019, Wiley. HAADF‐STEM images of D) Ru/1T‐MoS_2_ (left) and Ru/2H‐MoS (right). Schematics of the crystallographic difference between 2H and 1T‐MoS_2_. E) NRR and F) HER activities of 2H‐MoS_2_, 1T‐MoS_2_, Ru/1T‐MoS_2_, and Ru/2H‐MoS_2_ in 10 mm aqueous HCl. Reproduced with permission.^[^ ^111^ ^]^ Copyright 2019, American Chemical Society.

The synergistic effect between Fe_3_O_4_ and Au in Au–Fe_3_O_4_ NPs results in a higher eNRR activity than that of Fe_3_O_4_, Au, and Au@Fe_3_O_4_ (core@shell) NPs.^[^ ^103^ ^]^ Au–Fe_3_O_4_ and Au@Fe_3_O_4_ NPs synthesized through a simple one‐pot wet‐chemical method have similar morphologies (**Figure** **7**A). The Au 4*f* XPS spectra (Figure 7B) show that the ratio of Au^3+^ to Au^0^ in Au–Fe_3_O_4_ NPs is higher than that in the other catalysts. Additional Au─O bonds in the Au–Fe_3_O_4_ NPs facilitate their strong binding to the intermediates, thereby enhancing the eNRR. The Au–Fe_3_O_4_ NPs provide an NH_3_ yield rate of 21.42 µg mg_cat_^−1^ h^−1^ and 10.54% FE at −0.2 V versus RHE (Figure 7C). The enhanced N_2_ chemisorption peak of Au–Fe_3_O_4_ NPs in the N_2_ temperature‐programmed desorption curves demonstrates the unique role of the heterojunction structure in the eNRR performance. Theoretical calculations indicate that the N_2_ to *N_2_H transition occurs on the Fe atoms, while Au optimizes the adsorption energy of NRR intermediates, making the NRR path on Au–Fe_3_O_4_ along an energetically favorable process. A metastable amorphous material with dangling bonds is known to be more catalytically active than its crystalline counterpart.^[^ ^109^ ^]^ Li et al. reported that the eNRR activity of amorphous Au NPs supported on bisubstrate CeO*_x_*–RGO (a‐Au/CeO*_x_*–RGO) was higher than that of their crystalline counterparts. This hybrid catalyst with a low Au loading (1.31 wt%) was prepared via coreduction. CeO*_x_* facilitates the conversion from crystalline to amorphous Au, whereas RGO serves as a support for the uniform dispersion of the a‐Au NPs. The high concentration of unsaturated coordination sites in the metastable amorphous material consequently improves the chemical reactivity with N_2_ molecules. Subsequently, Au sub‐nanoclusters (≈0.5 nm) embedded on TiO_2_ with a loading of 1.542 wt% were demonstrated to be promising eNRR catalysts.^[^ ^110^ ^]^ The size of the Au clusters significantly influences the formation of NH_3_ from N_2_. Moreover, in an acidic electrolyte, the partially positively charged Au active centers, through the Au─O─Ti bond, preferentially adsorb electroneutral N_2_ than H^+^ cations, resulting in an efficient eNRR.

To achieve high catalytic activity, Suryanto et al. reported a strategy for the polymorphic engineering of NPs.^[^ ^111^ ^]^ Ru clusters were decorated on the 1T and 2H polymorphs of MoS_2_ to form Ru/1T‐MoS_2_ and Ru/2H‐MoS_2_ hybrids, respectively. Figure 7D depicts STEM images of the hybrid and crystal structures of 2H and 1T‐MoS_2_. The catalytic activity and selectivity of Ru‐decorated semiconducting 2H‐MoS_2_ toward eNRR are higher than that of metallic 1T‐MoS_2_ (Figure 7E). Also, evaluation of HER performances revealed the crystal structure dependent eNRR/HER relationship. (Ru)/1T‐MoS_2_ showed better HER performances than those of (Ru)/1H‐MoS_2_ (Figure 7F). The eNRR performances show that Ru functions as N_2_ active site and 1H‐MoS_2_ provides protons to form NH_3_ at the interface of Ru/1H‐MoS_2_. Thus, a high eNRR activity can be obtained by tuning the HER kinetics of MoS_2_ via polymorphic engineering. The synergistic effects between different metals in intermetallic compounds enable them to be potential candidates for the eNRR.^[^ ^112^, ^113^, ^114^ ^]^ Pd‐, Au‐, and Ru‐based alloys are commonly used as intermetallic compounds. According to Tong et al., body‐centered cubic PdCu NPs, obtained by annealing their face‐centered cubic (FCC) counterparts, are more favorable for the eNRR because the energy barrier is lower than that of FCC PdCu. The phase transformation of PdCu positively shifts its d‐band center and improves its ability to bind nitrogen molecules. DFT calculations reveal that the strong d–d coupling between Pd and Cu enhances the transfer activity, enabling an efficient eNRR.^[^ ^115^ ^]^

### 3.2 Strategies for Enhancing INRR Performance

#### 3.2.1 Engineering SEI Layers on the Li Cathode

As discussed in Section 2.2, the reaction is initiated after a highly reductive potential is applied to form a Li metal surface from Li^+^.^[^ ^116^ ^]^ The highly reductive environment can be detrimental to electrolytes, proton sources, or other electrolyte components, which electrochemically disintegrate into the SEI, and this has been extensively investigated in Li‐ion battery studies. The SEI is considered a “passivation layer” that exclusively allows Li cations to reach the electrode surface and acts as an ionic conducting separator to prevent further electrolyte decomposition or undesirable Li dendrite formation.^[^ ^117^, ^118^ ^]^ The SEI promotes the stability and performance of the Li‐mediated NRR.^[^ ^77^, ^119^ ^]^ Moreover, it can control the relative diffusion rates of Li^+^, H^+^, and N_2_ ($r_{{\mathrm{Li}}^{+}}$, $r_{H^{+}}$, $r_{N_{2}}$), which are pivotal variables to affect the rate and selectivity. Despite considerable investigation, the formation mechanism and structure of the SEI in NRR remain unclear.

**Figure 8:**
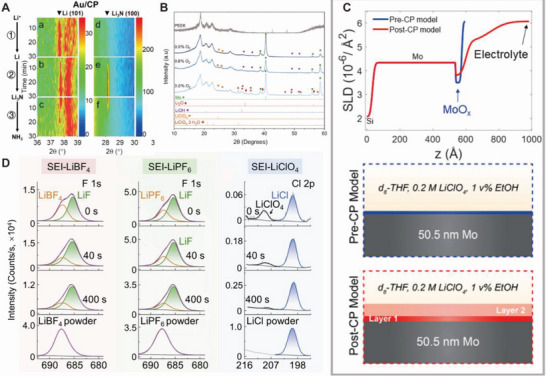
Investigation of SEI structure via spectroscopic analysis. A) Color map of operando‐XRD measurement during Li‐mediated NRR at Au/CP electrode to monitor the dynamic evolution of lithium‐containing intermediates. Reproduced with permission.^[^ ^120^ ^]^ Copyright 2020, Wiley VCH. B) XRD diffractograms of postelectrolysis electrode (Mo foil) with variation of O_2_ mol%. Reproduced with permission.^[^ ^118^ ^]^ Copyright 2021, American Association for the Advancement of Science. C) Scattering‐length density profiles of before (Pre‐CP) and after (Post‐CP) NRR electrolysis on Mo electrodes demonstrating the formation of SEI layers and corresponding schemes of Pre‐CP and Post‐CP model, respectively. Reproduced with permission.^[^ ^121^ ^]^ Copyright 2022, American Chemical Society. D) Dept‐profiling XPS spectra of the Cu electrodes depending on the electrolyte materials, which shows the formation of different SEI layers. Reproduced with permission.^[^ ^124^ ^]^ Copyright 2022, Elsevier.

Several researchers have performed real‐time spectroscopic analyses to investigate the composition of the cathode surfaces during catalysis. Through the in situ XRD analysis of Au‐coated carbon fiber paper, Gao et al. confirmed that Au facilitates Li‐ion reduction (**Figure** **8**A).^[^ ^120^ ^]^ They reported that the spontaneous initiation of nitrogen splitting to Li_3_N and protonation to NH_3_ occurred immediately after the rate‐determining Li‐ion reduction. A predominant peak of Li (101) was observed at ≈37°–38° only for Au on carbon paper (Au/CP) electrode, indicating that Au assists in rapid Li^+^ reduction kinetics. Only the Au/CP electrode exhibits a gradual increase in the Li_3_N (100) peak, whereas the pristine CP electrode presents no signal.

Although NRRs typically employ pure N_2_ gas to maximize the N_2_ content in the feed, practical applications may involve O_2_ inputs from air or other sources. Li et al. discovered that adding small amounts of O_2_ improves the efficiency and stability of the eNRR.^[^ ^118^ ^]^ To analyze these effects of O_2_ on the SEI layer, the changes in the phase and surface composition were monitored using XRD and X‐ray photoelectron spectroscopy (XPS). The XRD data (Figure 8B) revealed that as the O_2_ level increased to 3 mol%, various oxidized products were derived from the working electrode, owing to parasitic side reactions and oxygen reduction. Interestingly, the 0.8 mol% O_2_ atmosphere only showed a distinct peak in N 1s XPS spectra, corresponding to the Li_3_N layer, without forming any undesirable oxidized product. Through microkinetic modeling, the oxygen‐induced changes in the SEI layer were found to delay Li^+^ diffusion, while optimizing the availability of N_2_ and H^+^ in the system.

Blair et al. investigated the dynamic behavior of the SEI structure and its composition via in situ neutron reflectometry.^[^ ^121^ ^]^ Scattering‐length density profiles of the cathode–electrolyte interface during Li‐NRR on Mo thin‐film electrodes were obtained before and after chronopotentiometry (CP). Figure 8C shows that only the Mo‐oxide surface layer is detected in the pre‐CP electrode, whereas additional components from the interfacial layer are observed in the post‐CP electrode. The secondary layer after CP was considered the decomposed electrolyte product (SEI layer).

Li et al. further attempted to reveal the effects of Li‐containing electrolyte salts through theoretical modeling and depth‐profiling XPS. Three different salts (LiBF_4_, LiPF_6_, and LiClO_4_) were investigated using XPS spectra, based on the etching time (Figure 8D). SEI layer formation was confirmed after a short reductive electrolysis in all three cases. For example, a LiF‐enriched SEI layer was detected when using LiBF_4_. They reported that a LiBF_4_ electrolyte induced the formation of a compact and uniform SEI layer, resulting in homogeneous Li plating and enhanced NRR efficiency.^[^ ^124^ ^]^

#### 3.2.2 Electrode Engineering

**Figure 9:**
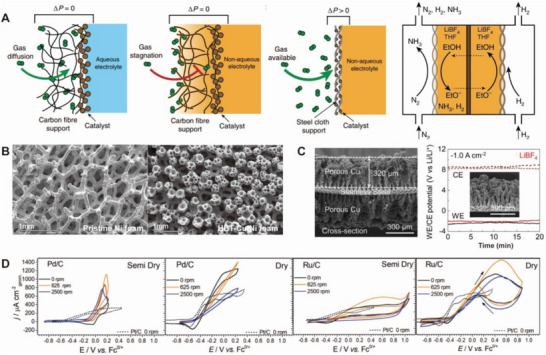
Electrode engineering strategies in INRR. A) Schematic illustration of structures of gas diffusion electrode in aqueous/porous carbon, nonaqueous/porous carbon (flooded), and nonaqueous/steel cloth. Proposed overall eNRR schemes with NRR at cathode and HOR on anode. Reproduced with permission.^[^ ^76^ ^]^ Copyright 2020, Springer Nature. B) SEM images of pristine Ni foam and HBT‐Cu/Ni foam. Reproduced with permission.^[^ ^122^ ^]^ Copyright 2021, American Chemical Society. C) Cross‐sectional SEM images of porous Cu electrode and its chronopotentiometry profile with LiBF_4_ electrolyte. Reproduced with permission.^[^ ^124^ ^]^ Copyright 2022, Elsevier. D) Cyclic voltammetry curves of various metal anode materials in H_2_ saturated 0.1 m LiNTf_2_/THF solution with varying humidity conditions, Pd/C [H_2_O] = 9 mm (semi dry) and 0.6 mm (dry), Ru/C [H_2_O] = 5 mm (semidry) and 0.4 mm (dry), respectively. Reproduced with permission.^[^ ^125^ ^]^ Copyright 2022, American Chemical Society.

Effective mass transport of N_2_ to the electrode is crucial to facilitate the splitting of the dinitrogen bonds. However, owing to the low solubility of N_2_ in nonaqueous solvents, the reactants are less accessible to the catalysts. To address the issue, Lazouski et al. designed an effective gas–liquid contacting gas diffusion electrode (GDE) in nonaqueous solvents.^[^ ^76^ ^]^ **Figure** **9**A depicts the typical aqueous media/GDE system, which allows direct gas contact with the catalysts on hydrophobic carbon supports. On the other hand, with the nonaqueous solvent, the carbon supports are inevitably wetted, and the catalyst is flooded, which results in limited N_2_ transport and a low mass‐transfer‐limited current. Lazouski et al. developed a catalyst‐deposited stainless‐steel cloth, and a pressure gradient was maintained across the cloth. As shown in Figure 9A, N_2_ was able to freely approach the catalysts without passing through the liquid phase, significantly improving NRR reaction rates and selectivity.

In the Li‐NRR scheme, the working electrode provides active and stable sites for Li deposition. Tsuneto et al. tested several metals as working electrodes; Mo and Cu electrodes have typically been used in Li‐mediated NH_3_ synthesis because they do not form alloys with Li,^[^ ^123^ ^]^ thus ensuring the reversible formation of the Li layer on the electrode. Figure 9B displays the SEM images of pristine Ni foam (left) and Cu electrodeposited on Ni foam using the hydrogen bubble template (HBT) method (right).^[^ ^122^ ^]^ Li et al. reported that a high‐surface‐area Cu electrode fabricated via the HBT method successively demonstrated a current density of −100 mA cm_geo_^−2^ at 20 bar N_2_. Owing to the porous Ni foam and the HBT method, a high ECSA of 66.5 ± 7.1 cm^2^ was obtained with a geometric surface of 0.5 cm^2^. The HBT–Cu electrode exhibited a NH_3_ yield rate (46.0 ± 6.8 nmol s^−1^ cm_geo_^−2^) superior to that of the pristine Cu electrode. Norskov & Chorkendorff group further attempted to use stainless steel mesh to prepare porous Cu via the HBT method.^[^ ^124^ ^]^ Figure 9C shows cross‐sectional SEM images of the porous Cu electrode on the stainless steel mesh. The three porous Cu samples prepared with different deposition times (15 s, 1 min, and 5 min) exhibited different specific capacitances and ECSA. The 5 min Cu sample exhibited the highest performance (15.4 mF cm_geo_^−2^; 308 cm^2^), resulting in 95% ± 3% FE at a current density of −100 mA cm_geo_^−2^ under 20 bar N_2_. Additionally, stable ammonia production was achieved on the porous Cu/steel electrode with the use of LiBF_4_ as the electrolyte condition.

In the typical Li‐NRR scheme, the anodic reaction has not been thoroughly investigated compared to the cathodic reaction. Recently, several studies have been conducted to replace the anodic reaction with a hydrogen oxidation reaction (HOR) by introducing an external hydrogen feed.^[^ ^125^, ^126^ ^]^ In this scheme, the anodic reaction can contribute to the efficiency of the Li‐NRR by providing protons to the catholyte. To determine the catalytic properties of the HOR, Hodgetts et al. investigated the poisoning on the catalyst surface by varying the water content.^[^ ^125^ ^]^ The authors hypothesized that poisoning of the surface from the adsorption of the electrolyte species and organic solvent oxidation products can be overcome by choosing proper electrode materials and water contents. Figure 9D displays the cyclic voltammetry curves of Pd/C and Ru/C catalysts under semidry and dry conditions (semidry: [H_2_O] = ≈4–10 mm/dry: [H_2_O] = ≈0.4–0.6 mm). The HOR current densities of Ru/C at 0.4 V versus Fc^0/+^ are higher than those of Pt/C under dry conditions. Both catalysts exhibit favorable HOR activities, but are plagued by low stability owing to water poisoning. Accordingly, bimetallic PtRu/C HOR catalysts were suggested with optimal catalytic activity and water tolerance under HOR.

#### 3.2.3 Electrolyte Engineering

**Figure 10:**
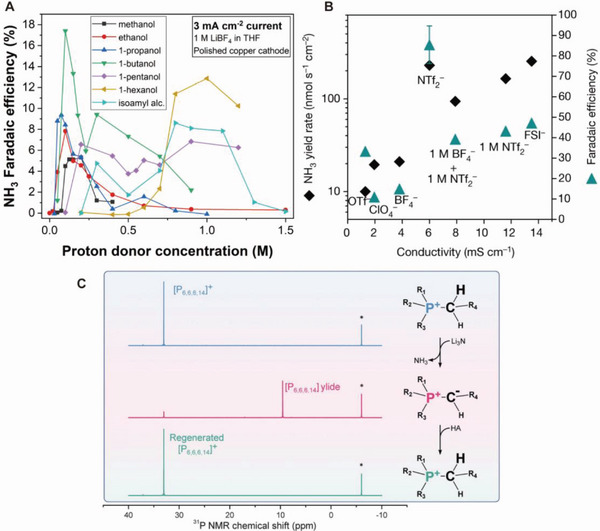
Electrolyte component engineering in INRR. A) Faradaic efficiency toward NH_3_ with the variation of proton donor species and concentration. Reproduced with permission.^[^ ^77^ ^]^ Copyright 2022, American Chemical Society. B) NH_3_ yield rate and Faradaic efficiency versus conductivity of various electrolyte species. Reproduced with permission.^[^ ^128^ ^]^ Copyright 2022, Springer Nature. C) ^31^P NMR spectra of phosphonium proton carrier during deprotonation and reprotonation cycle. [P_6,6,6,14_]^+^ cation peak at 32.9 ppm diminished after the reaction with Li_3_N and a new peak at 9.3 ppm emerged, which was assigned to the deprotonated ylide form. The acetic acid treatment restored the peak at 32.9 ppm. Reproduced with permission.^[^ ^129^ ^]^ Copyright 2021, American Association for the Advancement of Science.

The recent efforts for engineering SEI compositions and electrode materials have been discussed thus far. Other approaches have been also suggested for enhancing the Li‐NRR performance.^[^ ^127^ ^]^ Manthiram et al. examined diverse proton donors to reveal the effect of the proton donor structure on the transport kinetics in Li‐NRR.^[^ ^77^ ^]^ **Figure** **10**A shows that slight changes in the proton donor structure significantly impact the selectivity toward NH_3_. The electrolytes that form similar SEI species to that derived from THF can result in higher FEs owing to the enhanced SEI permeability, which directly affects the diffusion rates of N_2_ and the proton donor to the electrode surface. Here, n‐butanol exhibits the highest NH_3_ FE and forms an SEI similar to that obtained from THF.

Apart from adjusting the proton donors, the Li‐containing electrolyte, another key component in Li‐NRR, is being investigated. Recently, Du et al. reported almost 100% FE with 2 m LiNTF_2_.^[^ ^128^ ^]^ According to the plot of FE and NH_3_ yield rates against the conductivity of various Li compounds (Figure 10B), LiNTF_2_ has the highest NH_3_ yield rate and FE. The dense ionic assembly formed at the electrode–electrolyte interface suppresses electrolyte decomposition and provides high Li^+^ transference numbers.

In INRR scheme, the “proton shuttle” can play an important role to deliver the protons generated from the anode to the lithium cathode for the hydrogenation of lithium nitride. The proton shuttle can reversibly and reproducibly operate the catalytic cycle without participating in the side reaction during catalysis. In this vein, Suryanto et al. suggested reproducible phosphonium cation trihexyltetradecylphosphonium ([P_6,6,6,14_]^+^) as a proton shuttle.^[^ ^129^ ^]^ The authors demonstrated the reversibility of the salts with ^31^P NMR (Figure 10C) analysis. When the [P_6,6,6,14_][eFAP] (([P_6,6,6,14_]‐phosphonium tris(pentafluoroethyl)trifluorophosphate) solution was treated with excess Li_3_N, [P_6,6,6,14_]^+^ cation peak at 32.9 ppm was diminished and a new peak at 9.3 ppm emerged, corresponding to the deprotonated ylide form. Recovery of the proton shuttle was achieved by adding a proton source.

## 4 Summary and Outlook

**Figure 11:**
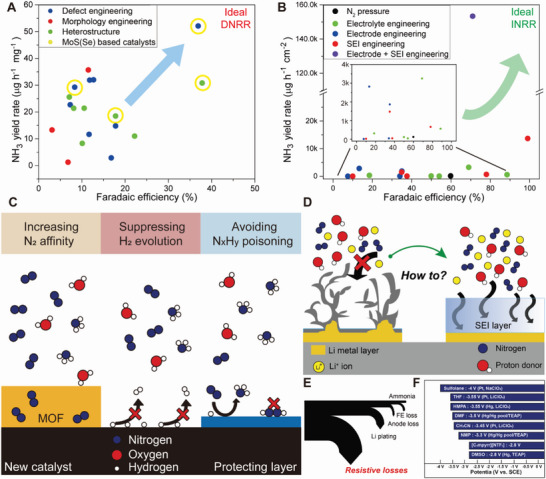
Summary and challenges of eNRR. Plots of ammonia yield and Faraday efficiency of previously reported studies in DNRR, and INRR. Each reactivity point is further categorized based on engineering strategies. A) Catalyst design strategies for DNRR; Engineering of the defect (blue), morphology (red), heterostructure (green), and Mo‐based chalcogenide catalysts (yellow). B) Strategies for INRR; Engineering of the electrode (blue), SEI (red), electrolyte (green), the integrated strategy of electrode and SEI layers (purple). C) Remaining tasks in DNRR; Increasing N_2_ affinity, suppressing H_2_ evolution, and avoiding N*_x_* H*_y_* poisoning. Remaining tasks in INRR. D) achieving the uniformity of the SEI layer. E) Minimizing resistive losses, and F) optimizing the solvent‐electrolyte composition.

In this review, we have highlighted the recent progress in electrochemical nitrogen reduction catalysis for NH_3_ production. We defined two approaches in terms of the reaction mechanisms: DNRR and INRR. DNRR follows a conventional electrocatalysis scheme and thus the development of an effective catalyst governs the overall performance of the system. We also discussed the currently adopted mechanistic pathways of nitrogen reduction and introduced several strategies for developing efficient catalysts. The DNRR requires considerable overpotentials from −0.7 to −0.2 V based on the electrolyte conditions. Among the reported catalysts, Mo‐based chalcogenide catalysts showed good DNRR performances, as shown in **Figure** **11**A; and Table S1 (Supporting Information). Although Bi‐based catalysts showed a decent performance,^[^ ^130^ ^]^ the FE and NH_3_ yields of most electrocatalysts are still low for practical ammonia production due to the competing HER and the low solubility of N_2_.

On the other hand, the performance of the Li‐mediated INRR is superior in terms of FE and partial current. Notably, the FE approached 100% in recent studies (purple circle in Figure 11B; and Table S2, Supporting Information).^[^ ^124^ ^]^ It demonstrated a higher possibility of realizing the electrochemical Haber–Bosch process. However, INRR also has several limitations to be addressed. The Li/Li^+^ redox couple requires a high reductive potential, which results in energy inefficiency and undesirable side reactions such as solvent/electrolyte decomposition and SEI layer formation.^[^ ^131^ ^]^ In addition, the use of the scarce and expensive Li metal may limit the applicability of this method. Therefore, considering the current achievement and remaining issues, we provide the key challenges to be addressed and future research directions, as follows.

### 4.1 Direct Nitrogen Reduction Reaction

#### 4.1.1 Enhancing the Availability of N_2_

Because DNRR is a surface‐confined reaction, enhancing N_2_ affinity via surface modifications such as atomic configuration (or functionalization) and maximization of electrode surface area would be a useful strategy (Figure 11C). Hybrid structures, such as metal–organic frameworks composed of the catalyst and functional agent, may effectively capture N_2_ on the catalyst surface and accelerate N_2_ dissolution based on Le Chatelier's principle.^[^ ^132^ ^]^ Also, since eNRR in the practical membrane electrode assembly (MEA) device is operated with humidified N_2_ gas due to the activation of membrane electrolyte, fine‐tuning the pore structures of the catalyst layer like in the case of the fuel cell would be an effective approach to enhancing the eNRR performances by expediting gas phase reactant supply and product removal in the MEA.

#### 4.1.2 Efficiently Suppressing the HER

As discussed in Section 3, most reports introduced enhanced DNRR performance, but most faradaic currents predominantly came from the HER.^[^ ^48^, ^58^ ^]^ As protons are obtained from water in aqueous electrolytes, modifying the wettability of the catalyst surface can effectively control proton availability. Generally, in electrochemistry, hydrophilic carbon (on hydrophobic gas diffusion layers) is used as a supporting material. The thing is that the carbon electrode typically shows marginal HER performance at the eNRR potentials or promotes the HER by adsorbing water molecules on their surfaces. Therefore, tuning the surface wettability should be considered when designing DNRR catalysts.^[^ ^133^, ^134^ ^]^ Furthermore, DNRR in nonaqueous electrolytes should be considered for enhancing the DNRR currents by stoichiometrically matching the number of protons for DNRR and minimizing HER currents.

#### 4.1.3 Preventing Surface Poisoning from Hydrazine or NH_3_

Similar to CO and CO_2_ poisoning in electrochemical hydrogen and methanol oxidation reactions, surface poisoning from species such as CO, NH_3_, and N_2_H_2_ would considerably affect the DNRR performance because surface passivation may induce catalyst deactivation and degradation. For example, a graphene layer coating on the catalyst surface may effectively minimize the poisoning by controlling the surface adsorption behaviors of these species (for example, CO poisoning during hydrogen oxidation).^[^ ^135^ ^]^

#### 4.1.4 In Situ and Operando Studies to Gain Mechanistic Insights

To gain a deeper understanding of reaction mechanisms and to elucidate the dynamic behavior that occurs at the reaction interface, it is essential to use a combination of electrokinetic, in‐situ, ex‐situ spectroscopy, and computational studies. In recent years, in situ techniques such as differential electrochemical mass spectrometry (DEMS), surface‐enhanced infrared absorption spectroscopy (SEIRAS), surface‐enhanced Raman spectroscopy (SERS), and attenuated total reflection infrared spectroscopy (ATR‐IR) have been utilized in the study of ammonia oxidation, the reverse reaction of nitrogen fixation. These techniques have enabled the identification of N‐containing intermediate species formed on active sites.^[^ ^136^, ^137^, ^138^ ^]^ By using a combination of DEMS and cyclic voltammetry, as well as DEMS and SEIRAS, it is possible to obtain information on adsorbed intermediates during the reaction. Combining these in situ instruments can provide valuable insights for the advance of catalyst designs.

### 4.2 Indirect Nitrogen Reduction Reaction

#### 4.2.1 Engineering the Architecture of the SEI Layer

Engineering the architecture of the SEI layer: As discussed previously, Li plating frequently induces the growth of an undesired SEI layer owing to solvent/electrolyte decomposition (Figure 11D, left). The permeability of the SEI layer is a crucial factor in the Li‐mediated NRR, because the diffusion of N_2_ and proton donor molecules to the electrode can be significantly affected by the structure of the SEI layer. Modifying the electrolyte composition and concentration can influence the formation kinetics of the SEI layer; an optimized current density might prevent undesirable dendritic Li growth and result in a uniform layer. Constructing an artificial SEI layer from electrolyte additives might suppress the randomized growth of the Li and SEI layers. According to previous studies on Li batteries, adding a fluorinated polysulfonamide electrolyte or SnF_2_ stabilizes the SEI composition and inhibits dendrite formation. Similar strategies may also be used for optimizing the SEI.

#### 4.2.2 Overcoming the Inferior Energy Efficiency

Li^+^ reduction to Li metal requires a high negative potential accompanied by solvent degradation and oxidation at the anode, which results in extremely high electrolyte resistance (typically 1000–1200 ohm); therefore, the energy efficiency is low (below 3%) for NH_3_ production. Decreasing the solution resistance can possibly increase energy efficiency. Another promising strategy involves coupling the INRR with hydrogen oxidation or other sacrificial anodic reactions to protect the solvent molecules. Furthermore, modifying the electrolyte composition can decrease unwanted energy loss. Other factors that need to be addressed include FE or NH_3_ loss during synthesis and limited cell lifetime (Figure 11E).

#### 4.2.3 Ideal Electrolyte Composition for eNRR

Most studies involving Li‐NRR have typically used THF because of its broad negative potential window. Figure 11F shows several candidate solvents that could be stable at highly negative potentials. Ionic liquid‐based electrolytes could be alternative candidates as stable N_2_‐soluble solvents.^[^ ^139^ ^]^ In Section 3.2.3, we discussed that the structures of the solvent and proton donor determine the diffusivity of the reacting species. Therefore, various electrolyte/solvent/HA components must be investigated to determine the optimal combination for an efficient NRR.

#### 4.2.4 Effective Proton Delivery

For continuous ammonia production, effective proton transport to the lithium nitride layer is particularly crucial. High‐performance continuous synthesis for commercialization necessitates a consistent stream of proton sources during the reaction. So far, hydrogen oxidation or anodic oxidation of proton donors have served as proton sources.^[^ ^76^, ^77^, ^125^ ^]^ Facile accessibility of proton to cathode has been tried by several groups^[^ ^129^, ^140^ ^]^ introducing proton shuttle concepts. Additionally, in order to facilitate proton transport, it is important to regulate the solvation of protons in nonaqueous media. In this vein, Kamlet–Taft solvent parameters can provide valuable guidelines to find out the optimal composition of the electrochemical system.^[^ ^141^ ^]^ Polarizability (π*) and hydrogen bond acceptor ability (*β*) of the solvents and proton donors would have a strong correlation for the transport of proton to SEI layers. Controlling SEI structure and proton transport will be the key to maximizing the overall efficiency of the Li‐mediated NRR.

In conclusion, we believe that eNRR would provide numerous possibilities to expand the applications of heterogeneous electrocatalysts and also to achieve global carbon neutrality. At present, the eNRR is technically at the initial stage, and distinct merits and drawbacks have been suggested for the two eNRR routes. In particular, the Li‐mediated INRR reaches ampere‐scale currents of NH_3_ production, which potentially can be comparable to the commercialized electrolysis system, in near future. We hope that, with this review, researchers in the fields of nanomaterials and electrochemistry gain significant insights into the development of eNRR‐related materials and technologies.

## Conflict of Interest

The authors declare no conflict of interest.

## Author Contributions

H.J., S.S.K., and S.V. contributed equally to this work. Conceptualization, H.J., S.S.K., and K.J.; Writing—original draft, H.J., S.S.K., S.V., and K.J.; Figure sets—J.L.; Writing—review & editing, H.J., K.L., and K.J.; funding acquisition, K.J. and K.L.; supervision; K.J and K.L.

## Supporting information

Supporting Information
