# Supplementary material for: Electrochemical Nitrogen Fixation for Green Ammonia: Recent Progress and Challenges
Source: Adv Sci (Weinh). 2023 Jun 8;10(23):2300951. doi: 10.1002/advs.202300951 (PMC10427382; doi:10.1002/advs.202300951)
Supplement: Supplementary file 1 — Supporting Information [file ADVS-10-2300951-s001.pdf]

ADVANCED SCIENCE
Open Access

Supporting Information

for *Adv. Sci.*, DOI 10.1002/advs.202300951

Electrochemical Nitrogen Fixation for Green Ammonia: Recent Progress and Challenges

Haneul Jin, Suyeon S. Kim, Sandhya Venkateshalu, Jeseok Lee, Kwangyeol Lee and Kyoungsuk Jin**

Supporting information

Electrochemical Nitrogen Fixation for Green Ammonia: Recent Progress and Challenges

Haneul Jin[1,†], *Suyeon S. Kim*[2,†], *Sandhya Venkateshalu*[2,†], *Jeseok Lee*[2], *Kwangyeol Lee*[2,*], *Kyoungsuk Jin*[2,*]

[1]Department of Energy and Materials Engineering, Dongguk University-Seoul, Seoul 04620, Republic of Korea

[2]Department of Chemistry and Research Institute of Natural Science, Korea University, Seoul, 02841, Republic of Korea

† *These authors contributed equally*

*Corresponding authors

Email: kylee1@korea.ac.kr (K. Lee)

Email: kysjin@korea.ac.kr (K. Jin)

Table S1. Table for catalysts for DNRR

Catalyst	Salt	Potential (V vs. RHE)	FE (%)	Yield rate	Main strategy for high ammonia yield	Ref.
W_2N_3	0.10 M KOH	−0.2	11.67 ± 0.93	11.66 ± 0.98 μg h^{-1} mg^{-1}	Defect	93
NNiO/CC	0.1 M $LiClO_4$	−0.5	7.30	22.7 μg h^{-1} mg^{-1}	Defect	90
C-Ti_xO_y/C	0.1M $LiClO_4$	−0.4	17.80	14.8 μg h^{-1} mg^{-1}	Defect	94
B-Graphene	0.05 M H_2SO_4	−0.5	10.80	9.8 μg h^{-1} cm^{-2}	Defect	88
CN/C	0.1 M HCl	−0.3	16.80	2.9 μg h^{-1} mg^{-1}	Defect	92
$Cu_{1.81}S$	0.5M Na_2SO_4	−0.5	3.1 ± 0.3	13.3 ± 0.7 μg h^{-1} mg^{-1}	Morphology	101
Ir-$Cu_{1.81}S$	0.5 M Na_2SO_4	−0.6	2.4 ± 0.1	18.2 ± 0.8 μg h^{-1} cm^{-2}	Defect	97
PdRu	0.1 M HCl	−0.1	1.53	25.92 μg h^{-1} mg^{-1}	Morphology	95
MoS_2 nanoflower	0.1 M Na_2SO_4	−0.4	8.34	29.28 μg h^{-1} mg^{-1}	Defect	99
Layered Ni_3S_4	0.1M K_2SO_4	−0.2	6.8 ± 3.3	1.28 μg h^{-1} mg^{-1}	Morphology	96
Fe-MoS_2	0.1 M KCl	−0.2	31.6 ± 2	97.5 μg h^{-1} cm^{-2}	Defect	104
pAu/NF	0.1 M Na_2SO_4	−0.2	13.36	9.42 μg h^{-1} cm^{-2}	Morphology	98
SnO_2/RGO	0.1 M Na_2SO_5	−0.5	7.10	25.6 μg h^{-1} mg^{-1}	Heterostructure	109
CeO_x–RGO	HCl	−0.2	10.10	8.3 μg h^{-1} mg^{-1}	Heterostructure	111
Au/TiO_2	HCl	−0.2	8.11	21.4 μg h^{-1} mg^{-1}	Heterostructure	112
Fe-N,O doped C	0.1M HCl	−0.4	11.80	31.9 μg h^{-1} mg^{-1}	Defect	14
CuO/RGO	0.1 M Na_2SO_4	−0.75	3.90	1.8×10^{-10} mol cm^{-2} s^{-1}	Heterostructure	106
Au–Fe_3O_4	0.1 M KOH	−0.2	10.54	21.42 μg h^{-1} mg^{-1}	Heterostructure	105
MOS_2	0.01 M HCl	−0.15	17.60	$1.14\times10{-}^{10}$ mol cm^{-2} s^{-1}	Morphology	68
MoS_2/C_3N_4	0.1M HCl	−0.3	17.80	18.5 μg h^{-1} mg^{-1}	Heterostructure	110
PdCu	LiCl	−0.1	11.50	35.7 μg h^{-1} mg^{-1}	Morphology	115
B-Mo_2C	0.5 M K_2SO_4	−0.6	36.90	52.1 μg h^{-1} mg^{-1}	Defect	S1
Pd/PdO	0.1 M KOH	0.03	22.20	11.0 μg h^{-1} mg^{-1}	Heterostructure	6
Au/$MoSe_2$	0.1 M Na_2SO_4	−0.3	37.82	30.83 μg h^{-1} mg^{-1}	Heterostructure	S2
C_8-MoS_2-CP	0.1 M Na_2SO_4	−0.25	22.23	12.86×10^{-11} mol cm^{-2} s^{-1}	Morphology	30
Ni@Nb_2C	0.1 M KOH	−0.4	7.30	26.16 μg cm^{-2} h^{-1}	Heterostructure	28
Bi/K co-doped TiO_x	0.25 M $LiClO_4$	−0.8	12.71	32.02 μg h^{-1} mg^{-1}	Defect	S3
Fe(II)Cu(II)Fe(III)-LDH	0.1 M Na_2SO_4	−0.5	21.7 ± 1.8	33.1 ± 2.5 μg h^{-1} mg^{-1}	Defect	S4
TiO_2@N-C	0.1 M KOH	−0.1	2.46	5.302 μg h^{-1} cm^{-2}	Heterostructure	54
N,S co-doped $Ti_3C_2T_x$	0.05 M H2SO4	−0.55	6.60	34.23 μg h^{-1} mg^{-1}	Defect	S5

Table S2. Table for INRR performances

Electrode	Salt	Electrolysis condition	N_2 pressure	Highest FE	Partial current density (mA cm^{-2})	Yield rate (μg h^{-1} cm^{-2})	Main strategy for high ammonia yield	Ref.
Cu wire	0.2M Li(OTf) / THF / 0.18 M EtOH	CP (-1.0 mA cm^{-2})	50 atm	59.8%	0.598	122.4	N_2 pressure	72
Steel	LiOH-LiCl	-4 V (vs. $Li^{0/+}$)	1 bar	88.5%	177	565.2	Electrolyte	73
Ni	1M $LiClO_4$	CP (-5 mA cm^{-2})	—	49.9%	2.4965	115.2	Electrolyte	74
Cu foil	1M $LiBF_4$ / THF / 0.1M EtOH	CP (-8 mA cm^{-2})	1 bar	18.5 ± 2.9%	1.48	313.92	Electrolyte	75
Mo foil	0.2M $LiClO_4$ / THF / 0.18M EtOH	-9 V (vs. RHE)	1 bar	7.5 ± 1.1%	—	13.68	Electrode	S6
Mo rod	0.5M $LiClO_4$ / THF / 0.18M EtOH	CP (-0.8 mA cm^{-2})	1 bar	10%	0.1	25.56	SEI	82
Mo foil	0.2M $LiClO_4$ / THF / 0.18M EtOH	CP (-2.0 mA cm^{-2})	10 bar	37.6 ± 2.0 %	0.752	52.92	SEI	80
Pt/Stainless-steel cloth	1M $LiBF_4$ / THF / 0.1M EtOH	CP (-25 mA cm^{-2})	10 sccm	35 ± 5%	8.75	1864.8	Electrode	76
Au/CP	0.2M $LiClO_4$ / THF / 0.18M EtOH	-10.0 V (two-electrode)	10 sccm	34.0 ± 4.5%	—	48.96	Electrode	122
Stainless-steel cloth	1M $LiBF_4$ / THF / 0.11M EtOH	-7.3 V (vs. Ag/Ag^+)	5 sccm	34.9 ± 1.7%	6.98	1476	SEI	79
Cu disk	0.2M $LiBF_4$ / 0.1M [P6,6,6,14][eFAP]	CP (-22.5 mA cm^{-2})	19.5 bar	69 ± 1%	15.525	3250.8	Electrolyte	131
Mo foil	0.3M $LiClO_4$ / THF / 0.18M EtOH	CP (-4 mA cm^{-2})	20 bar (0.8 mol% O2)	78.0 ± 1.3%	3.12	662.4	SEI	120
Cu wire	0.2M LiOTf / THF / 0.086M EtOH	-1.1V (vs. $Li^{0/+}$)	15 bar	54 ± 7%	—	48.96	Electrolyte	S7
HBTCu/Ni foam	2M $LiClO_4$ / THF / 0.18M EtOH	CP (-100 mA cm^{-2})	20 bar	13.3 ± 2.0%	13.3	2818.8	Electrode	124
Ni wire	2M $LiNTF_2$ / THF / 0.1M EtOH	-0.55V (vs. $Li^{0/+}$)	15 bar	99 ± 2 %	—	13680	SEI	130
Porous Cu /SS	2M $LiBF_4$ / THF / 0.17M EtOH	CP (-1.0 A cm^{-2})	20 bar	71 ± 3%	710	153360	Electode, SEI	126

References for supporting information

S1. Fan, B., Wang, H., Zhang, H., Song, Y., Zheng, X., Li, C., Tan, Y., Han, X., Deng, Y. & Hu, W. Phase Transfer of Mo_2C Induced by Boron Doping to Boost Nitrogen Reduction Reaction Catalytic Activity. *Adv. Funct. Mater.* **32**, 2110783 (2022).

S2. Chen, D., Luo, M., Ning, S., Lan, J., Peng, W., Lu, Y.-R., Chan, T.-S. & Tan, Y. Single-Atom Gold Isolated Onto Nanoporous $MoSe_2$ for Boosting Electrochemical Nitrogen Reduction. *Small* **18**, 2104043 (2022).

S3. Liang, J. *et al.* Ion-exchange-induced Bi and K dual-doping of TiO_x in molten salts for high-performance electrochemical nitrogen reduction. *J. Energy Chem.* **69**, 26-34 (2022).

S4. Kong, Y., Kong, H., Lv, C. & Chen, G. Engineering Reductive Iron on a Layered Double Hydroxide Electrocatalyst for Facilitating Nitrogen Reduction Reaction. *Adv. Mater. Interfaces* **9**, 2102242 (2022).

S5. Zeng, Y., Du, X., Li, Y., Guo, Y., Xie, Y., Huang, J., Rao, G., Lei, T., Gong, C., Wang, X., and Sun, B. (2021) Synergistic performance of nitrogen and sulfur co-doped Ti3C2TX for electrohydrogenation of N_2 to NH_3. *J. Alloys Compd.*, **869**, 159335.

S6. Andersen, S.Z., Čolić, V., Yang, S., Schwalbe, J.A., Nielander, A.C., McEnaney, J.M., Enemark-Rasmussen, K., Baker, J.G., Singh, A.R., Rohr, B.A., Statt, M.J., Blair, S.J., Mezzavilla, S., Kibsgaard, J., Vesborg, P.C.K., Cargnello, M., Bent, S.F., Jaramillo, T.F., Stephens, I.E.L., Nørskov, J.K., and Chorkendorff, I. (2019) A rigorous electrochemical ammonia synthesis protocol with quantitative isotope measurements. *Nature*, **570** (7762), 504–508.

S7. Cherepanov, P. V., Krebsz, M., Hodgetts, R.Y., Simonov, A.N., and Macfarlane, D.R. (2021) Understanding the Factors Determining the Faradaic Efficiency and Rate of the Lithium Redox-Mediated N2Reduction to Ammonia. *J. Phys. Chem. C*, **125** (21), 11402–11410.
